# A New Bioactive Glass/Collagen Hybrid Composite for Applications in Dentistry

**DOI:** 10.3390/ma12132079

**Published:** 2019-06-28

**Authors:** Devis Bellucci, Roberta Salvatori, Jessica Giannatiempo, Alexandre Anesi, Sergio Bortolini, Valeria Cannillo

**Affiliations:** 1 Dipartimento di Ingegneria Enzo Ferrari, Università di Modena e Reggio Emilia, Via Vivarelli 10, 41125 Modena, Italy; 2 SMECHIMAI, Università di Modena e Reggio Emilia, Largo del Pozzo 71, 41125 Modena, Italy; 3 CHIMOMO, Università di Modena e Reggio Emilia, Largo del Pozzo 71, 41125 Modena, Italy

**Keywords:** bioactive glass, composites, collagen, cell tests, biocompatibility

## Abstract

Bioactive glasses (BGs) are currently employed in a wide range of medical and dentistry applications by virtue of their bone-bonding ability. The incorporation of BGs into a collagen matrix may be used to combine the regenerative potential of these materials with the specific biological advantages of collagen. However, most of the collagen/BG composites reported in the literature are scaffolds and there is a lack of moldable putties or injectable systems. Here, granules of an innovative BG containing strontium and magnesium were mixed with collagen and PEG to obtain a putty (BGMS/C) suitable for dental applications. For the sake of comparison, granules of 45S5 Bioglass^®^, the gold standard among BGs, were used to prepare a 45S5/collagen putty. Both the composites were evaluated in vitro with respect to murine fibroblasts. The materials showed an excellent biocompatibility, making them interesting for possible applications in dentistry and reconstructive surgery. Moreover, BGMS/C seems to stimulate cell proliferation.

## 1. Introduction

Bioactive glasses (BGs) are, basically, non-crystalline ceramics able to bond living tissues and stimulate new tissue growth while dissolving over time; these properties make them valuable candidates for tissue engineering applications. Initially designed to fill bone defects, BGs progressively expanded their biomedical suitability towards a wide range of clinical purposes, especially thanks to the capability of specific BG compositions such as the 45S5 Bioglass^®^ to bind both to hard and soft tissues [1]. Recent research has revealed other intriguing properties of BGs, such as an antibacterial effect, angiogenesis-stimulating action, desensitization and remineralization efficacy, and potential applications in contact with soft tissues [2,3]. On the other hand, BGs suffer from some drawbacks (e.g., high tendency to crystallize during thermal treatments, poor mechanical strength and high brittleness) which limited their widespread use. In order to tackle these disadvantages, BGs as granules or powders have also been mixed with a wide range of polymers, aiming at producing hybrid composites with mechanical and biological performance tailored for a specific clinical application [4]. Natural polymers, and in particular collagen, are of the utmost importance in the biomaterials field. Collagen is the main structural protein in the extracellular matrix and it can be extracted from various tissue sources, being the most abundant protein in mammals. By virtue of its superior biocompatibility, biodegradability, low immunogenicity, and wound-healing properties, collagen is the protein of choice; in particular, several collagen-based composites containing BG inclusions have been reported, with specific focus on their application in bone regeneration [5]. In our study, an innovative bioglass (BGMS10) in the form of granules was mixed with collagen and PEG, specifically employed as a binder, in order to obtain a putty (BGMS/C) suitable for oral and dental applications including periodontal pockets, bone defects, and mucosal injury; PEGs are a class of viscous polymers with different molecular weight, widely used in the pharmaceutical and cosmetic industries as excipients, solubilizers, binding agents, etc. [6]. BGMS10, containing strontium and magnesium, is particularly promising by virtue of its ultrahigh crystallization temperature and bioactivity [7]. A 45S5/collagen putty (45S5/C), with the same proportions of glass, collagen, and PEG as that of BGMS/C, was prepared for comparison. The biological performance of both putties was tested with respect to murine fibroblasts. None of the prepared composites was cytotoxic; moreover, the BGMS10/C looks particularly promising, being able to foster cell proliferation.

## 2. Materials and Methods 

### 2.1. Composites’ Preparation

An innovative bioactive glass (BGMS10, composition, in mol.%: 2.3 Na_2_O; 2.3 K_2_O; 25.6 CaO; 10.0 MgO; 10.0 SrO; 2.6 P_2_O_5_; 47.2 SiO_2_) was produced by a classical melt-quenching route [8]. The bioactive glass was fully characterized in terms of thermal and physical properties in a previous work [7]. Briefly, the raw powder reagents (from Carlo Erba Reagenti, Milano, Italy) were melted at 1450 °C in a Pt crucible in air. The molten bioglass was rapidly quenched in water to obtain a frit, subsequently left to dry at 110 °C for 12 h. Finally, the frit was ground and sieved to obtain granules with grain size between 150 µm and 250 µm. Such granules then observed in a SEM (ESEM Quanta 2000, FEI Co., Eindhoven, The Netherland) equipped with EDS spectroscopy (Inca, Oxford Instruments, Abington, UK) to perform qualitative compositional analyses.

Proper amounts of commercial hydrolyzed collagen (Boya Cosmetics Ltd., Hong Kong, China) and glass granules were mixed with PEG-4000 (Farmalabor, Assago, Italy) to prepare the following set of composites:

- BGMS/C: 31 wt.%BGMS10, 43 wt.% collagen, 26 wt.% PEG;
- 45S5/C: 31 wt.% 45S5, 43 wt.% collagen, 26 wt.% PEG.

The samples were sterilized by gamma-radiation before biological tests.

### 2.2. Biocompatibility Tests

NIH/3T3 (murine embryo fibroblast) cells were used to investigate the biocompatibility in vitro, according to International Standards [9,10]. The biological performance of the produced putties was tested both through direct contact, where the cells are seeded directly onto the materials, and indirect contact, in order to evaluate possible cytotoxic effects of the composites’ eluates. NR uptake, MTT, and BrdU assays were employed to investigate cell viability and proliferation, respectively. 

NIH/3T3 cells were grown in DMEM with 100 μg/mL penstreptomycin and 10% (v/v) FBS (Invitrogen). DMEM only and DMEM with 0.45% phenol solution were used as negative (CTRL-) and positive (CTRL+) controls, respectively. 

The NR assay [11] is based on the ability of healthy cells to incorporate within their lysosomes the neutral red, a supravital dye. The cells were cultured in direct contact with the putties for 24 h. Both the materials and the culture medium were then removed and 150 μL of NR solution (N6264 Sigma, Germany) were added. After incubation (3 h) the NR solution was removed and 1.5 mL of extraction solution (ethanol/acetic acid) was added to all wells and left to incubate (10 min). The amount of NR dye incorporated by the cells was evaluated by spectrophotometry (Diode Array HP 8452A, Hewlett-Packard, Palo Alto, CA, USA) at 540 nm. CTRL- and the CTRL+ were used as references. The cell’s morphology was observed in an optical microscope (Leitz, Germany) after 24 h of incubation.

MTT is a colorimetric test used to investigate the cell viability after exposure to the samples’ eluates (i.e., indirect contact). It is based on the activity of mitochondrial enzymes in metabolic active cells, which reduce the tetrazolium salt MTT to a purple formazan, whose amount is proportional to the number of living cells [12]. Cells were grown in 96-well plates and then incubated with the materials’ eluates for 24 h. 80 μL of MTT labeling solution was then added to each well. After 4 h, 1 mL of dimethylsulfoxide was added to solubilize the formazan crystals. The amount of the formazan generated was quantified by spectrophotometry (Multiscan RC by Thermolab system, Helsinki, Finland) at 540 nm. 

The BrdU test is used to quantify the incorporation of 5-bromo-2-deoxyuridine in place of thymidine in the replicating DNA of proliferating cells, with the aim to evaluate cell proliferation. Cells were grown in 96-well culture plates and exposed to the materials’ extracts for 24 h. 10 μL/well of BrdU labelling solution (Cell Proliferation ELISA, BrdU, Roche Diagnostics, Basel, Switzerland) were then added and left to incubate (2 h); the labelling culture medium was subsequently removed and FixDenat solution was added to fix cells and denature DNA. After the solution removal, samples were incubated with an antibody conjugated to peroxidase (anti-BrdU-POD) which binds to the BrdU incorporated into the newly synthesized cellular DNA. Finally, the amount of the reaction product is quantified by spectrophotometry (Multiscan RC by Thermolab system, Helsinki, Finland) at 370 nm. 

## 3. Results 

**Figure 1:**
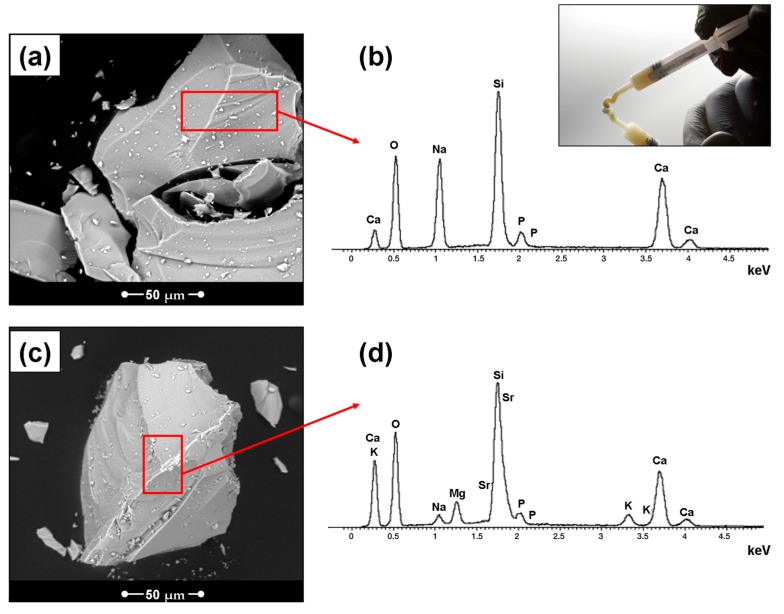
Morphological evaluation of the 45S5 (**a**) and BGMS10 (**c**) granules; (**b**,**d**) EDS results; inset: a BGMS/C putty syringe.

Figure 1 reports the morphological evaluation of the 45S5 and BGMS10 granules, together with their composition (by EDS analysis). As recently demonstrated [7], BGMS10 has an extraordinarily high crystallization temperature (932 °C) and a high in vitro bioactivity in simulated body fluid solution; its biocompatibility towards cells is here evaluated for the first time. There is a lack of literature focused on BGs containing both magnesium and strontium, despite the biological benefits of the following elements: Sr, indeed, can stimulate osteoblasts, inhibit osteoclasts in vitro, and accelerate bone-healing processes, while Mg increases bone cell adhesion, stimulates osteoblast proliferation, and differentiation, activates phagocytosis, etc. [11]. The grain size of the granules is appropriate to ensure adequate reactivity and feasibility of a composite suitable for clinical use, which basically means fluid enough to be syringeable and easy to use (i.e., malleable), but at the same time sufficiently viscous to limit its dispersion once injected. A BGMS/C putty syringe is shown in the inset of Figure 1. It should be stressed that although in recent years many collagen/bioglass composites were developed, most of them were porous scaffolds for bone tissue engineering. On the contrary, there is a lack of mouldable putties or injectable composites for dental applications. 

In this case, the specific biological responsiveness of the inorganic phase is responsible for the bioactive, regenerative, angiogenic, and antibacterial potential of the final composite, while the organic phase (collagen and PEG) assists the tissue healing mechanisms [13] and, in particular, makes the final putty mouldable and injectable, i.e., suitable for clinical use.

**Figure 2:**
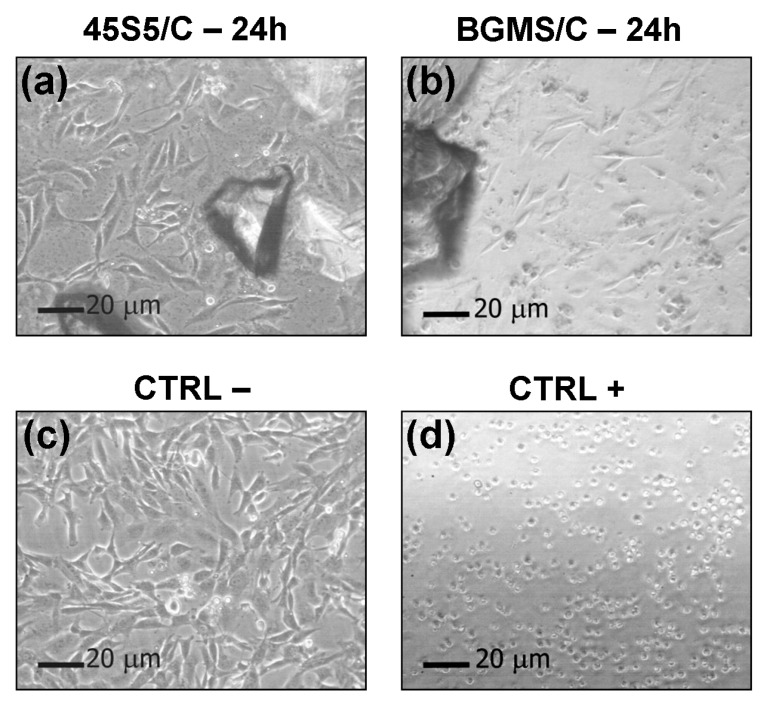
The morphology of NIH/3T3 cells after 24 h direct contact (optical microscope). (**a**) 45S5/C; (**b**) BGMS/C; (**c**) CTRL-; (**d**) CTRL+.

**Figure 3:**
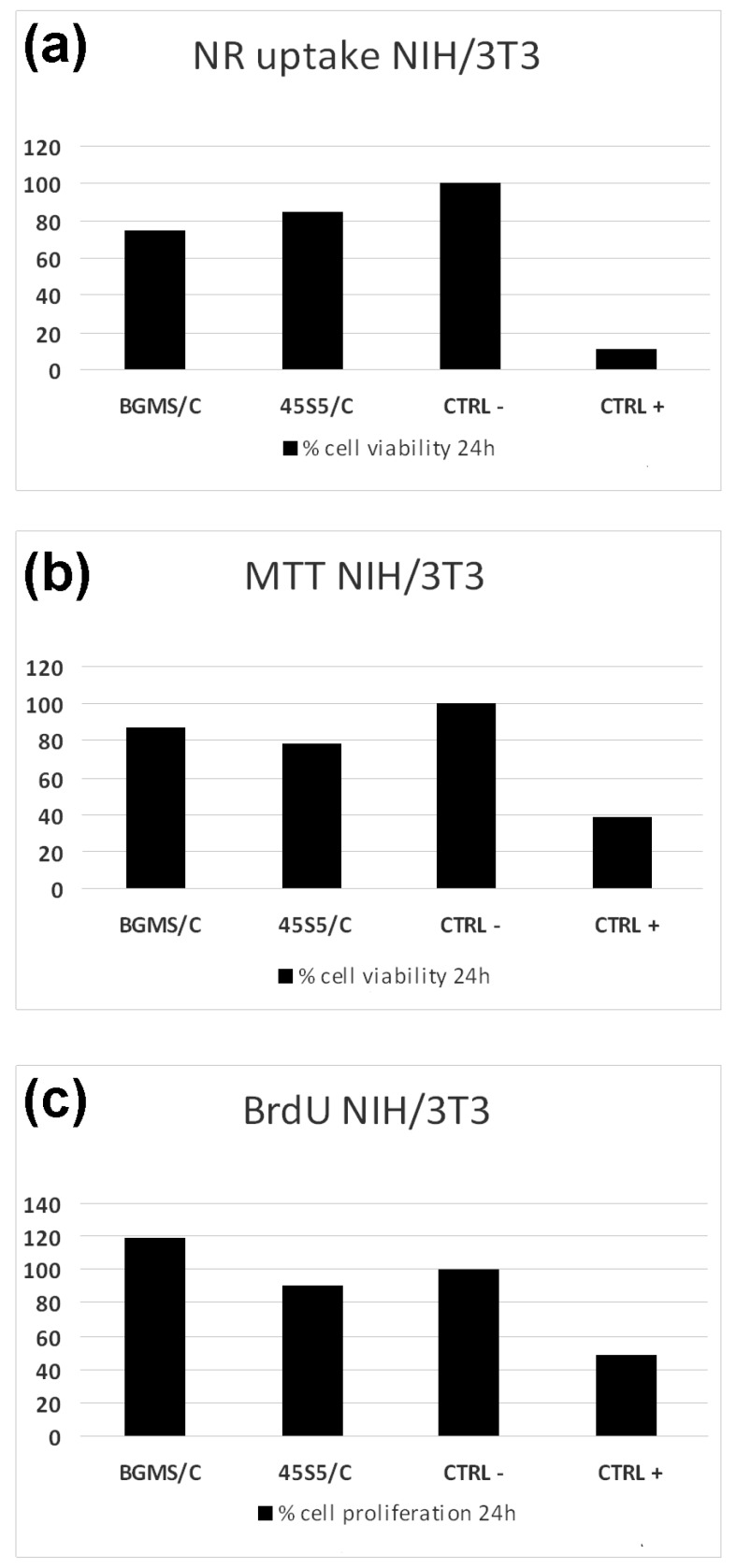
Results of the NR, MTT and BrdU tests for NIH/3T3 cells after 24 h. (**a**) NR uptake test; (**b**) MTT test; (**c**) BrdU test..

Figure 2 shows the optical micrographs of the NIH/3T3 cells after 24 h of direct contact with the putties. The cells’ morphology looks analogous to that of the cells in the CTRL-, thus excluding cytotoxic effects of the produced materials. These outcomes are confirmed by the results of both the NR and MTT viability tests in Figure 3. 

It should be noted that the use of both NR and MTT assays allows for the excluding of cytotoxic effects both by direct contact with the materials and their eluates. According to the BrdU results (Figure 3), the produced putties did not negatively affect the cell proliferation; moreover, the best results were obtained by the BGMS/C, which not only surpassed the 45S5/C, but also the CTRL-, thus showing a stimulating effect on cell proliferation. This fact may likely be ascribed to the presence of strontium in BGMS10, which stimulates the proliferation of several cell lines [14]. Moreover, in dentistry research strontium is supposed to improve the healing of bone and osseointegration of dental implants [15]. For these reasons, the new BGMS/C putty appears to be suitable for dentistry applications by virtue of its biocompatibility, possible stimulating effects, and malleability.

## 4. Conclusions

In this work, novel composites in the form of putty based on collagen, PEG, and an innovative BG containing strontium and magnesium, were produced. The biocompatibility of the materials was successfully confirmed towards NIH/3T3 cells, using both direct and indirect contact approaches, and results of the BrdU test revealed that BGMS/C putty stimulates NIH/3T3 cell proliferation after 24 h. This fact should be mainly ascribed to the specific BGMS10 composition, which contains strontium, magnesium, and lower amounts of alkaline oxides than the widely used 45S5. For these reasons and thanks to its malleability, the new BGMS/C putty looks particularly promising for possible applications in dentistry and reconstructive surgery.
